# Satellite cell content in Huntington’s disease patients in response to endurance training

**DOI:** 10.1186/s13023-019-1115-4

**Published:** 2019-06-11

**Authors:** Sandro Manuel Mueller, Violeta Mihaylova, Sebastian Frese, Jens A. Petersen, Maria Ligon-Auer, David Aguayo, Martin Flück, Hans H. Jung, Marco Toigo

**Affiliations:** 1 Department of Neurology, University Hospital Zurich, Zurich, Switzerland; 2 Research and Performance Center for Elite Athleticism, OYM, Lorzenparkstrasse 12, 6330 Cham, Switzerland; 3 Institute of Human Movement Sciences, ETH Zurich, Zurich, Switzerland; 4 Department of Orthopaedics, Laboratory for Muscle Plasticity, Balgrist University Hospital, University of Zurich, Zurich, Switzerland

**Keywords:** Muscle mass, Muscle wasting, Stem cell, Plasticity, Remodeling

## Abstract

**Background:**

Skeletal muscle wasting is a hallmark of Huntington’s disease (HD). However, data on myocellular characteristics and myofiber remodeling in HD patients are scarce. We aimed at gaining insights into myocellular characteristics of HD patients as compared to healthy controls at rest and after a period of increased skeletal muscle turnover.

**Methods:**

Myosin heavy chain (MyHC)-specific cross-sectional area, satellite cell content, myonuclear number, myonuclear domain, and muscle fiber type distribution were determined from vastus lateralis muscle biopsies at rest and after 26 weeks of endurance training in HD patients and healthy controls.

**Results:**

At the beginning of the study, there were no differences in myocellular characteristics between HD patients and healthy controls. Satellite cell content per MyHC-1 fiber (*P* = 0.014) and per MyHC-1 myonucleus (*P* = 0.006) increased significantly in healthy controls during the endurance training intervention, whereas it remained constant in HD patients (*P* = 0.804 and *P* = 0.975 for satellite cell content per MyHC-1 fiber and myonucleus, respectively). All further variables were not altered during the training intervention in HD patients and healthy controls.

**Conclusions:**

Similar skeletal muscle characteristics between HD patients and healthy controls at baseline suggested similar potential for myofiber remodeling in response to exercise. However, the missing satellite cell response in MyHC-1 myofibers following endurance training in HD patients points to a potential dysregulation in the exercise-induced activation and/or proliferation of satellite cells. In the longer-term, impaired myonuclear turnover might be associated with the clinical observation of skeletal muscle wasting.

## Main text

Huntington’s disease (HD) is a neurodegenerative disorder with symptoms encompassing motor, cognitive, and psychiatric dysfunctions. While the initial research on this disease focused mainly on the central nervous system, other organs and tissues, such as skeletal muscle, have additionally gained attention in the scientific literature in the past few years. For example, several studies in HD patients reported energy metabolism dysfunctions in skeletal muscle [1–4] and skeletal muscle pathology was well-described in mouse models [5–7]. Because skeletal muscle wasting is regarded as a hallmark of HD, skeletal muscle mass was also investigated in recent publications. In these studies, no differences in whole-body fat-free mass or lean mass between HD patients and healthy controls could be detected [8–11]. These findings challenge the notion of skeletal muscle wasting, at least in early- to mid-stage HD patients. Moreover, data on muscle fiber cross-sectional areas, satellite cells, myonuclei, and myofiber remodeling upon exercise in HD patients remain scarce.

**Fig. 1:**
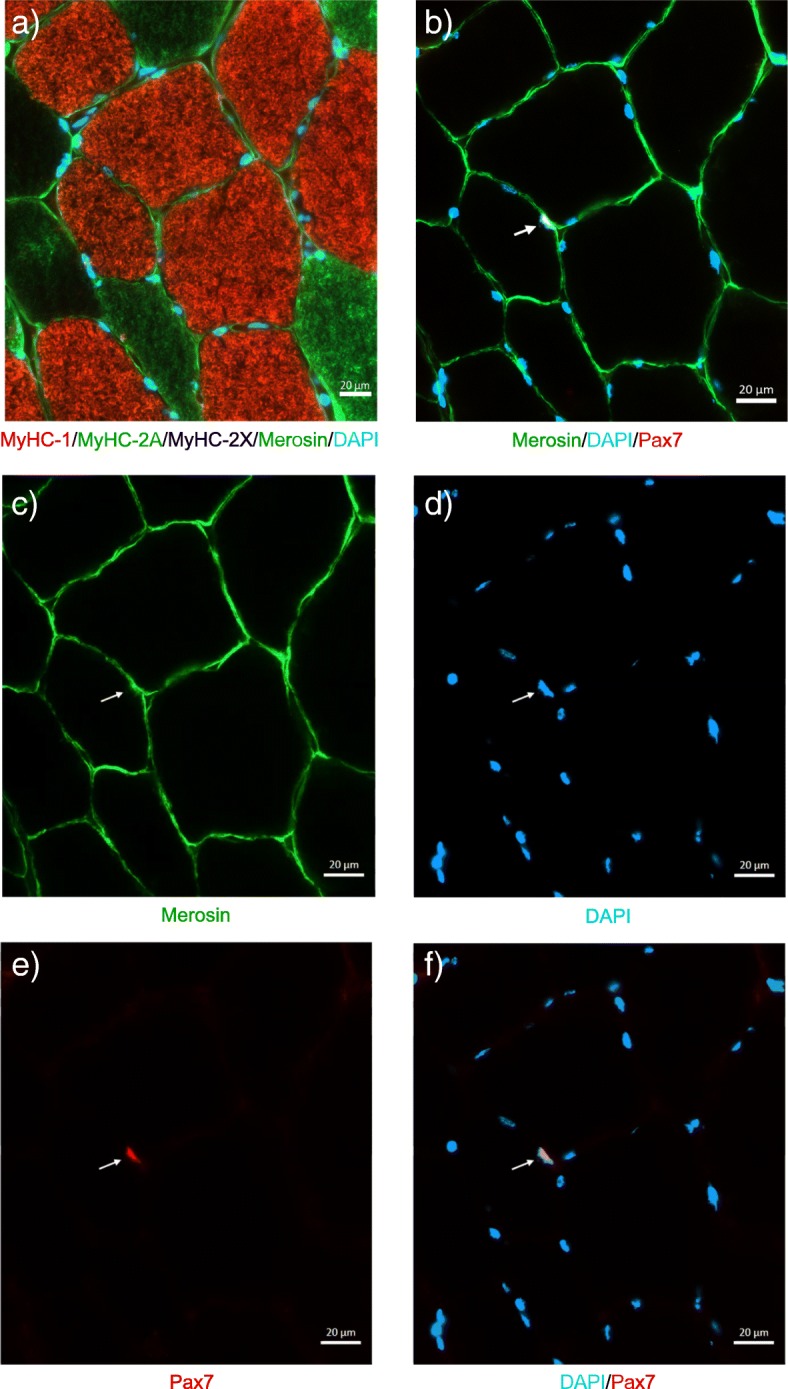
Representative images of fiber type-specific analyses of skeletal muscle satellite cell content. **a**) Myosin heavy chain (MyHC) isoforms stained as follows: MyHC-1 (red), MyHC-2A (green), MyHC-2X (unstained, black), **b**) co-occurrence of Pax7^+^ cell (red) with subsarcolemmal myonucleus (blue) and cell border line (green), **c**) merosin staining for cell borders (green), **d**) subarcolemmal myonuclei (blue), **e**) Pax7^+^ cell (red), **f**) co-occurrence of Pax7^+^ cell (red) with subsarcolemmal myonucleus (blue)

In the current preliminary study, we aimed at gaining insights into muscle fiber cross-sectional areas, satellite cell content, myonuclear content and myonuclear number of HD patients as compared to healthy controls at rest and after a period of increased skeletal muscle turnover. We reanalyzed muscle biopsy samples from a previous study [12]. Due to limitations in muscle tissue amount, we were not able to analyze all samples of the previous study. Muscle samples of nine HD patients (age: 51.7 ± 7.9 years, height: 1.74 ± 0.05 m, body mass: 75.9 ± 11.2 kg) and ten healthy control participants (age: 50.0 ± 6.7 years, height: 1.79 ± 0.05 m, body mass: 82.8 ± 13.0 kg) could be analyzed and the results thereof are presented in this report. According to the clinical assessments, the HD patients were classified as early- and intermediate-stage of disease. Myosin heavy chain (MyHC)-specific cross-sectional area, satellite cell content, myonuclear number, and myonuclear domain were determined from vastus lateralis muscle biopsies at rest and after 26 weeks of endurance training in HD patients and healthy controls (Fig. 1). An additional muscle biopsy sample was obtained from HD patients 26 weeks before beginning of the training intervention to assess alterations due to the natural disease progress.

**Fig. 2:**
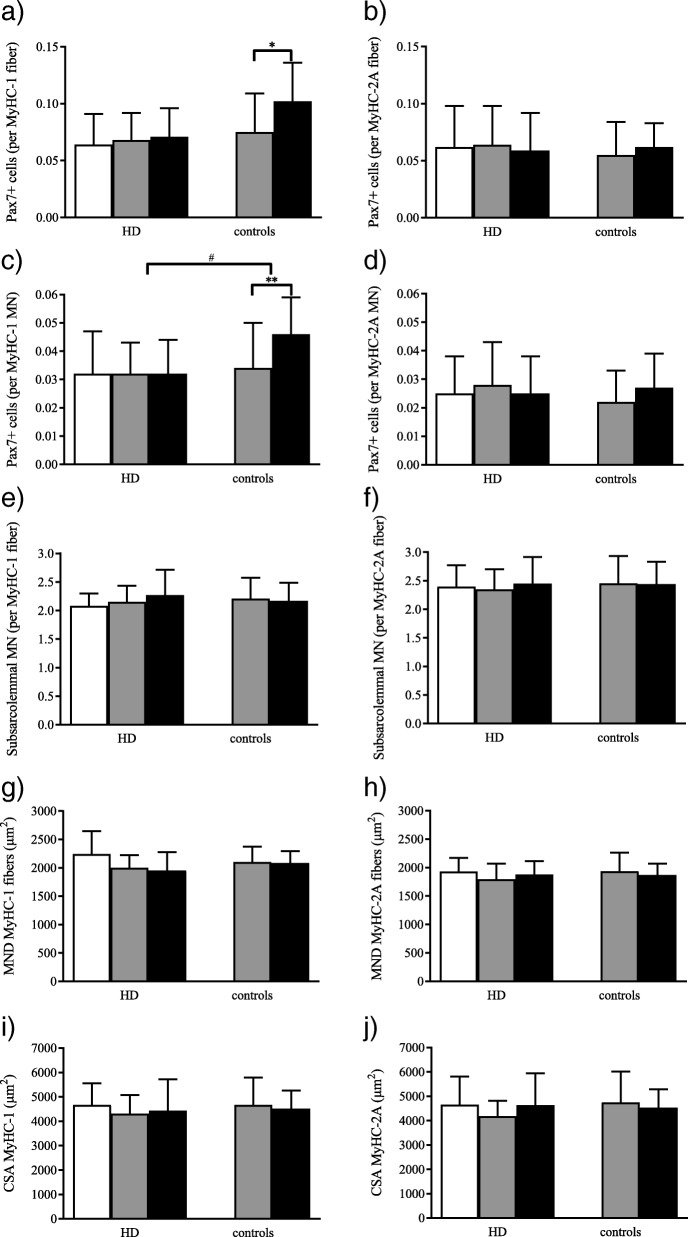
Baseline (white bars), pre- (grey bars) and post-training (black bars) values for HD patients (*n* = 9) and healthy control participants (*n* = 10) for satellite cell content **a**) per MyHC-1 fiber, **b**) per MyHC-2A fiber, **c**) per MyHC-1 fiber myonucleus (MN), **d**) per MyHC-2A fiber MN, for subsarcolemmal MN content in **e**) MyHC-1 fibers and **f**) MyHC-2A fibers, for myonuclear domains (MND) in **g**) MyHC-1 fibers and **h**) MyHC-2A fibers, for cross-sectional area (CSA) of **i**) MyHC-1 fibers and **j**) MyHC-2A fibers

At the beginning of the study, there were no differences in myocellular characteristics between HD patients and healthy controls (Fig. 2). Satellite cell content per MyHC-1 fiber (*P* = 0.014) and per MyHC-1 myonucleus (*P* = 0.006) increased significantly in healthy controls during the endurance training intervention, whereas it remained constant in HD patients (*P* = 0.804 and *P* = 0.975 for satellite cell content per MyHC-1 fiber and myonucleus, respectively). There was a significant group x time interaction for satellite cells per MyHC-1 myonucleus (*P* = 0.045). All further variables were not altered during the training intervention in HD patients and healthy controls. During the natural course observation period in HD patients, there were no fiber type-specific alterations in any variable.

Similar skeletal muscle characteristics between HD patients and healthy controls at baseline suggested similar potential for myofiber remodeling in response to exercise and indicated that these characteristics are not affected by pathological processes in the early- and mid-stage of the disease. This observation was further supported by the constant myocellular characteristics during the half-year natural course observation period. However, the missing satellite cell response in MyHC-1 myofibers following a half-year endurance training intervention in HD patients pointed to a potential dysregulation in the exercise-induced activation and/or proliferation of satellite cells. Due to the absence of new genetic material to replace damaged DNA, tissue remodeling might be impaired in subsequent years, resulting in an attenuation of myofiber regeneration. In conclusion, our result points to impaired myonuclear turnover in HD patients that might be associated with the clinical observation of skeletal muscle wasting in the longer-term.

## Data Availability

The datasets used and/or analyzed during the current study are available from the corresponding author on reasonable request.

## Abbreviations

HD: Huntington’s disease
MyHC: Myosin heavy chain
